# Virus-like Particles Produced in Plants: A Promising Platform for Recombinant Vaccine Development

**DOI:** 10.3390/plants13243564

**Published:** 2024-12-20

**Authors:** Eugenia S. Mardanova, Egor A. Vasyagin, Nikolai V. Ravin

**Affiliations:** Institute of Bioengineering, Research Center of Biotechnology of the Russian Academy of Sciences, 119071 Moscow, Russia

**Keywords:** plant, biofactory, vaccine, virus-like particle, transient expression

## Abstract

The capsid proteins of many viruses are capable of spontaneous self-assembly into virus-like particles (VLPs), which do not contain the viral genome and are therefore not infectious. VLPs are structurally similar to their parent viruses and are therefore effectively recognized by the immune system and can induce strong humoral and cellular immune responses. The structural features of VLPs make them an attractive platform for the development of potential vaccines and diagnostic tools. Chimeric VLPs can be obtained by attaching foreign peptides to capsid proteins. Chimeric VLPs present multiple copies of the antigen on their surface, thereby increasing the effectiveness of the immune response. Recombinant VLPs can be produced in different expression systems. Plants are promising biofactories for the production of recombinant proteins, including VLPs. The main advantages of plant expression systems are the overall low cost and safety of plant-produced products due to the absence of pathogens common to plants and animals. This review provides an overview of the VLP platform as an approach to developing plant-produced vaccines, focusing on the use of transient expression systems.

## 1. VLPs: From the Beginning to the Market

Virus-like particles (VLPs) have made enormous advances in the field of vaccinology over the last decades. Two structural proteins of hepatitis B virus (HBV), core (HBc) and surface (HBs) antigens, were among the first recombinant VLP candidates [1]. A prophylactic vaccine against hepatitis B virus in the form of virus-like particles formed by HBs antigen produced by yeast was the first recombinant vaccine for humans and has been used in public health since 1986. In 2006 and 2007, a cervical cancer vaccine based on VLPs formed by the L1 protein of the human papillomavirus, obtained in yeast or baculovirus expression systems, entered the market. The success of these two vaccines has accelerated progress in this field, leading to the approval of several VLP-based vaccines (see Supplementary Table S1), with others currently in various phases of clinical trials [1,2,3,4].

## 2. An Overview of the Transient Expression of Recombinant Proteins in Plants

Plant biofactories are promoted as a fast, efficient, and cost-effective alternative to bacteria and animal cells for the production of recombinant proteins [5]. Transgenic plants can be used to produce recombinant proteins, providing stable but typically low expression levels. In addition, generating transgenic plants is time-consuming. Nevertheless, Protalix Biotherapeutics (Israel) has produced the first FDA-approved pharmaceutical from plants. Currently, Protalix Biotherapeutics produces two drugs:

(i). Elelyso^®^ (taliglucerase alfa), produced in carrot cells for Enzyme Replacement Therapy (ERT), replaces the deficient glucocerebrosidase enzyme with a recombinant form of the protein in patients with a confirmed diagnosis of Type 1 Gaucher disease [6]. Elelyso was first approved in May 2012.
(ii). Elfabrio^®^ (pegunigalsidase alfa), produced in tobacco cells for ERT, replaces the deficient α-galactosidase-A enzyme with a recombinant form of the protein when administered through intravenous infusion. Elfabrio^®^, first approved in May 2023 [7], is identified for the treatment of patients with confirmed Fabry disease. Elfabrio was first approved in May 2023 [7].

Although edible plants such as potato, tomato, maize, soybean, rice, and carrot that produce vaccine proteins have been used to study the immunogenicity of, in particular, oral vaccines that induce a mucosal immune response [8,9,10], the use of food plants to produce pharmaceutical proteins such as vaccines has raised serious concerns among regulatory agencies [11].

An alternative to transgenic plants is the use of transient expression methods followed by purification of the target protein from plant biomass. Transient expression is achieved by transferring genes into plant cells using *Agrobacterium* infection. This process is known as agroinfiltration, whereby *Agrobacterium* enters the leaf interstitium and transfers its genetic material into plant cells, resulting in expression that is typically 100–10,000 times higher than that possible with stable transformation [12]. Transient expression does not depend on chromosomal integration and can be detected as early as 3 h after DNA delivery [13]. Transient expression allows the desired protein to be produced within weeks, which is especially important for vaccine development during epidemics. The company Medicago reported that it received the first doses of its COVID-19 vaccine candidate just 20 days after receiving a copy of the SARS-CoV-2 coronavirus RNA [14].

*Nicotiana benthamiana* plants are the most popular and widely used hosts for transient expression, offering a number of advantages, including non-food crop status, a high growth rate, and the availability of suitable and efficient vectors for efficient gene expression [15]. The standard tool for producing recombinant proteins in plants is binary plasmids containing an expression cassette within a transfer DNA (T-DNA) region, selection markers, and replication elements for *Escherichia coli* and *Agrobacterium tumefaciens*.

**Figure 1:**
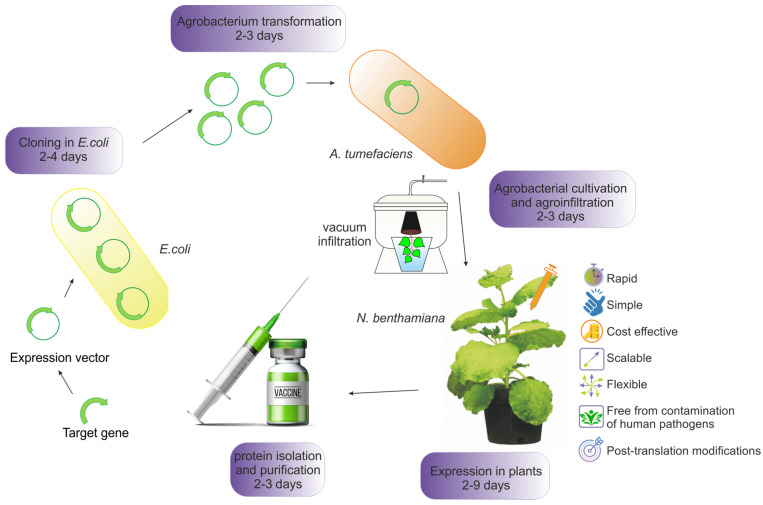
An overview of the transient expression of recombinant proteins in plants.

The general scheme of transient expression is presented in Figure 1. Expression vectors encoding the gene of interest are produced in *E. coli* and then transformed into *A. tumefaciens*, which can deliver its T-DNA region into plant cells. Suspensions of agrobacteria carrying expression vector regions are introduced by infiltration into leaves either manually using a syringe or by vacuum infiltration. Vacuum agroinfiltration can be used for large-scale protein production. This approach ensures the efficient penetration of agrobacteria into plant cells, which ultimately leads to a high level of synthesis of the target product [13]. After infiltration, the T-DNA of the expression vector is transferred into plant cells, and the target protein is produced in the plants for several days. The plant tissues are then collected, and the recombinant protein product is purified.

Efficient expression vectors are required to produce recombinant proteins in plants at high yields. High expression levels of recombinant genes in plants can be achieved using vectors based on plant virus genomes. Plant viruses are generally very efficient pathogens, using very compact genomes to produce their own proteins in host cells at high levels, up to 50% of the total cellular protein. Therefore, viral expression vectors have been developed that allow the efficient expression of recombinant proteins in plants.

The most popular plant expression vectors are based on RNA viruses such as tobacco mosaic virus (TMV), potato virus X (PVX), and cowpea mosaic virus (CPMV), as well as bean yellow dwarf virus, a DNA-containing geminivirus [13,16,17,18]. For example, Icon Genetics has developed the magnICON platform for protein production in plants based on the efficient assembly of DNA modules produced by recombination in plant cells [16,17,18,19].

A high-level expression system that utilizes elements of the replication machinery of bean yellow dwarf virus (BeYDV) has been developed [20,21]. This virus possesses a single-stranded circular DNA genome that replicates to very high copy numbers in the nuclei of infected cells [20].

George P. Lomonosoff’s group (John Innes Centre, UK) developed the pEAQ (easy and quick) expression system. This system involves inserting the gene to be expressed between the modified 5′-untranslated region (UTR) and 3′-UTR of cowpea mosaic virus (CPMV) RNA-2, which significantly increases the level of protein expression [22].

**Figure 2:**
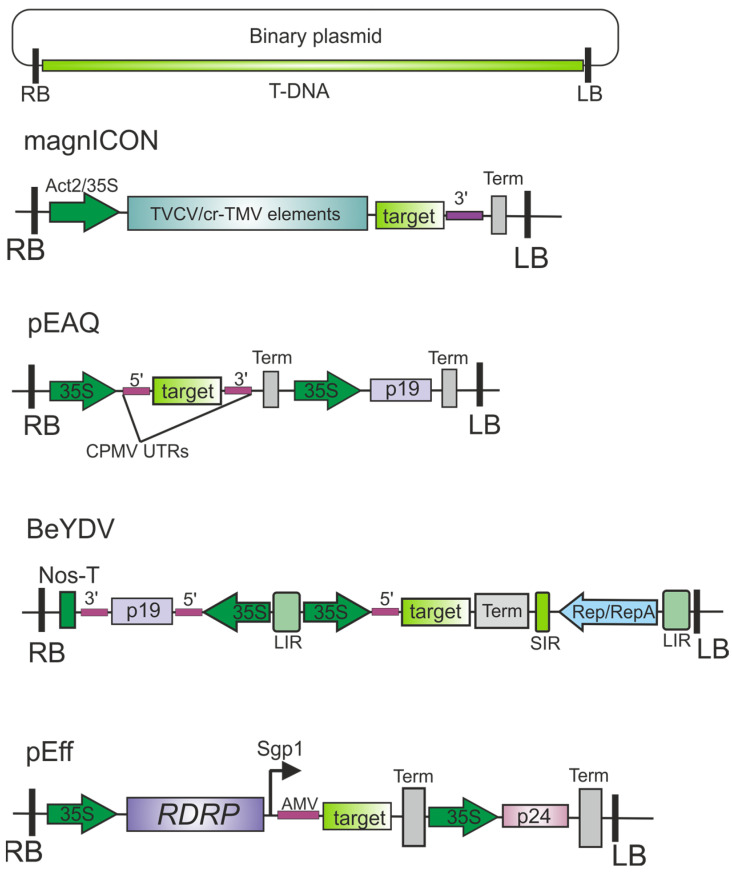
Some widely used plant transient expression systems. The T-DNA regions of plant expression vectors based on the genomes of turnip vein-clearing virus (TVCV) and crucifer-infecting TMV (cr-TMV) (magnICON), cowpea mosaic virus (CPMV) (pEAQ), bean yellow dwarf virus (BeYDV), and potato virus X (PVX) (pEff). RB and LB, the left and right T-DNA; *target*, gene of interest; Act2, Arabidopsis actin 2 promoter; 35S, the promoter of cauliflower mosaic virus RNA; Nos-T, the terminator of the *A. tumefaciens* nopaline synthase gene; Term, the terminator of transcription; *p19*, the gene of tomato bushy stunt virus silencing suppressor; LIR, long intergenic region; SIR, short intergenic region; Rep/RepA, replication proteins from BeYDV; *RDRP*, RNA-dependent RNA polymerase gene; Sgp1, the first promoter of subgenomic RNA of PVX; AMV, a translational enhancer from alfalfa mosaic virus; *p24*, the gene of grapevine leafroll-associated virus-2 silencing suppressor; 5′ and 3′, untranslated regions (of diverse origins).

**Figure 3:**
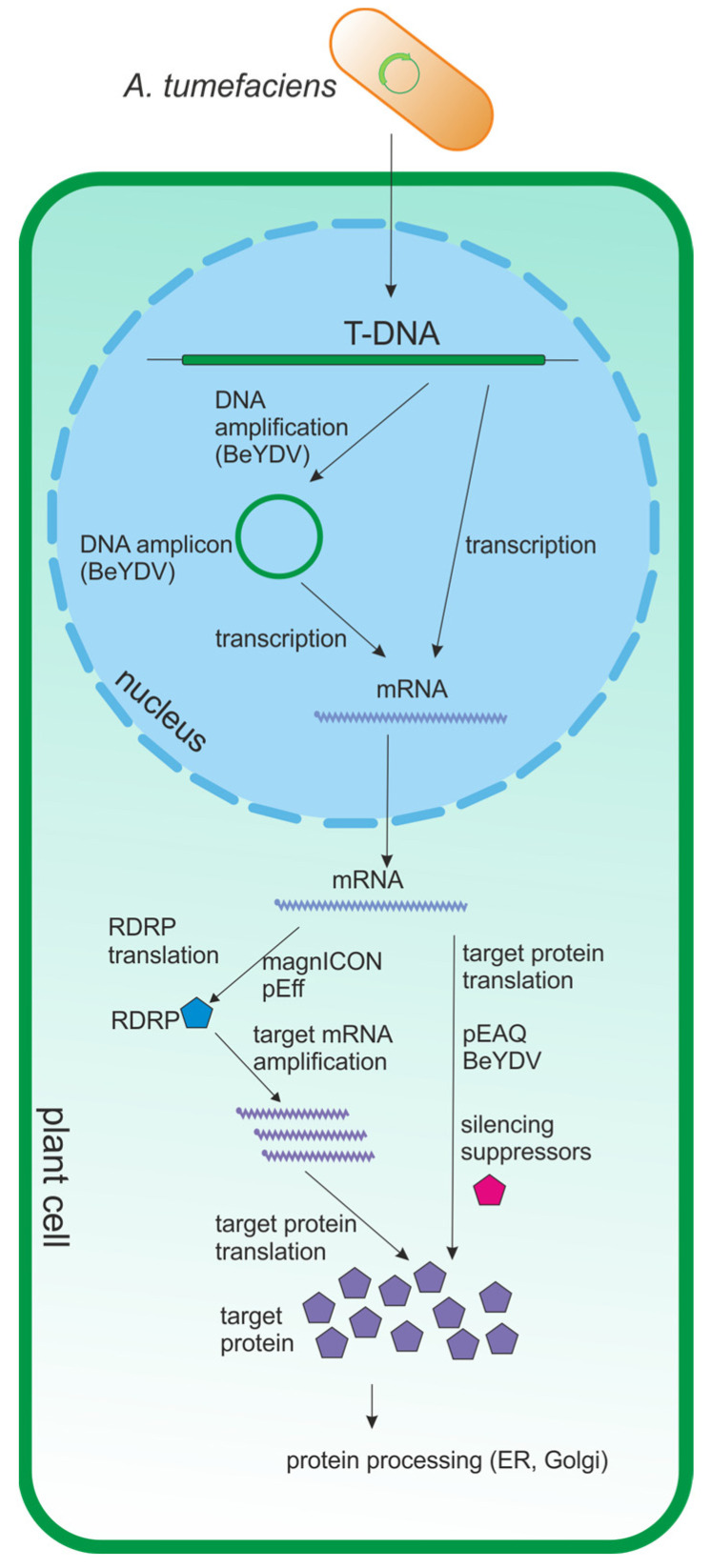
Scheme of transient expression in plant cells using viral expression vectors.

The self-replicating vector pEff, based on the genetic elements of potato virus X (PVX), particularly RDRP (RNA-dependent RNA polymerase) and the first promoter of subgenomic RNA, is widely used to produce various proteins in plants [23,24,25,26,27,28,29,30]. A general scheme of popular expression vectors and an overview of their mechanisms of action are presented in Figure 2 and Figure 3, respectively.

Different approaches can be used to optimize transient protein expression in *N. benthamiana*, including the use of chemical additives, heat shock, and the co-expression of genes known to suppress stress and gene silencing or stimulate cell cycle progression [31].

Post-transcriptional gene silencing (PTGS) is one of the most important factors limiting the efficiency of recombinant protein expression in plants. PTGS is a natural defense mechanism of plants against viruses and pathogens [32]. In turn, plant viruses have evolved to encode proteins able to suppress PTGS [33]. Suppression of PTGS provides increased yields of transiently expressed proteins in plants [33]. Different PTGS suppressors have been investigated [34], including p1 from rice yellow mottle virus [35], p10 from grapevine virus A [36], p19 from tomato bushy stunt virus [37], p21 from beet yellow virus [22,38], p24 from grapevine leaf roll-associated virus [39], p25 from potato virus X [40], p38 from turnip crinkle virus [41], 2b from cucumber mosaic virus [42], and HcPro from tobacco etch virus [43].

The PTGS suppressor gene can be contained in an expression vector maintained in a separate strain of *Agrobacterium* and introduced into plants together with a strain containing a vector encoding the target protein (co-agroinfiltration of two strains). An alternative method is to include the PTGS expression cassette into an expression vector encoding the target protein, which eliminates the need for co-infiltration. For example, p19 is part of pEAQ-HT [22], and p24 is part of the pEff vector [23] (Figure 3).

*Agrobacterium* is used to deliver the T-DNA of the expression vector into the plant cell nucleus. Transcription occurs in the nucleus, and BeYDV-based vectors undergo an additional step of DNA amplification by rolling circle replication in the nucleus (DNA amplification). Once the transcripts are released into the cytoplasm, they are either directly translated into target proteins (pEAQ, BeYDV) or undergo an RNA amplification step (magnICON, pEff) by RNA-dependent RNA polymerase (RdRP). The target recombinant proteins are then transported to the final subcellular compartments.

Research is also actively underway to increase the stability of proteins, direct them to various cell compartments, and create transgenic plants with “humanized” glycosylation pathways [44].

Despite progress in the creation of expression vectors and the development of transient expression methods, the expression level of recombinant proteins in plants is variable, often unpredictable, and depends on the properties of a particular protein. Another stumbling block remains the purification of proteins from plants. Despite these challenges, plant manufacturing is actively developing and has already achieved notable successes [45,46,47,48].

A number of biopharmaceutical companies and research laboratories are working on the transient expression of recombinant particles in plants [45]. The Canadian biotechnology company Medicago has been a pioneer and leader in the commercial development of plant molecular farming. Medicago has developed vaccines against seasonal influenza [49], pandemic influenza [50], and SARS-CoV-2 (based on the spike glycoprotein); the last one has been approved for use in Canada [49]. Though Medicago is no longer in business, many companies are engaged in plant molecular farming, such as BioApp (Seoul, Korea), Kbio (Owensboro, KY, USA), Denka/Icon Genetics (Tokyo, Japan), Thailand’s Baiya Phytopharm (Bangkok, Thailand), South Africa’s Cape Bio Pharms (Cape Town, South Africa), etc. [51,52].

## 3. Construction of Chimeric VLPs

Structural (capsid) viral proteins can retain the ability to self-assemble, in the absence of the viral genome, into VLPs that mimic the native virus and induce a protective immune response. VLPs stimulate innate immunity via Toll-like receptors (TLRs) and pattern recognition receptors (PRRs) due to the presence of multivalent structures, inducing a strong humoral response and enhancing the uptake, processing, and presentation by APCs through the MHC I and MHC II cross-presentation pathway due to the particulate nature of VLPs [2,53,54].

**Figure 4:**
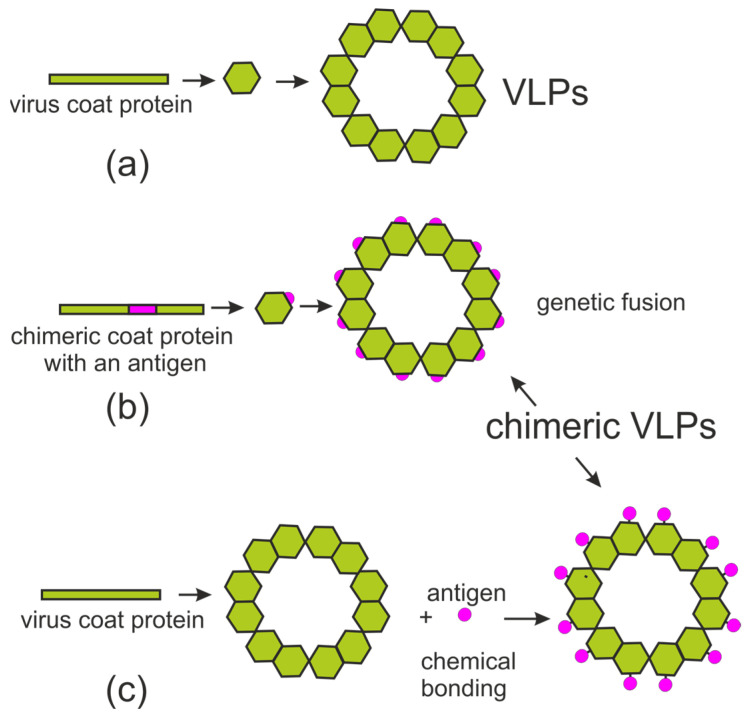
General scheme of chimeric VLP formation. (**a**) Native VLPs; (**b**) chimeric VLPs obtained by genetic fusion approach; (**c**) chimeric VLPs obtained by chemical crosslinking in vitro.

VLPs can be used not only as vaccines against the “parental” virus but also as carriers for an efficient presentation of foreign antigens to the immune system. To present foreign antigens, genetic fusion or chemical crosslinking techniques can be used for antigen display on VLPs (Figure 4). Genetic fusion is a simple method of incorporating an antigen that ensures its presence within a particle. Chimeric VLPs are produced by assembling capsid proteins with attached antigens. Particle formation is influenced by intermolecular chemical bonds and steric hindrances, which can be disrupted by antigen attachment. Thus, proper VLP assembly is highly unpredictable. This may lead to the formation of heterogeneous VLPs, and the antigens may not be optimally exposed on the surface; therefore, the immune responses generated against such antigens are often limited.

To overcome these difficulties, chemical antigen conjugation to VLPs can be used. Native VLPs and target antigens are synthesized separately. Next, the in vitro assembly of the two components is performed by either covalent or noncovalent binding, which links the target antigen to the surface of the preassembled VLP. Chemical crosslinking is a commonly used method to facilitate the binding of antigens to native VLPs [4], although binding of the antigen to the VLP can also be achieved through protein–protein interactions [55].

## 4. Plant-Derived Vaccines Based on VLPs Formed by Capsid Proteins of the Target Virus

### 4.1. Hepatitis B Virus

Hepatitis B virus core antigen (HBcAg) has attracted much attention as a potential basis for vaccines [56]. Pioneering work to produce VLPs formed by HBcAg was carried out in *N. benthamiana* using the MagnICON transient expression system [57]. HBcAg was produced in *N. benthamiana* leaves at levels up to 7% of total soluble protein or 2.4 mg per gram of fresh leaf biomass at 7 days post-infection (dpi). Plant-derived HBcAg assembled into VLPs and stimulated strong serum antibody responses in immunized mice. Such success has prompted research in this area. Later, it was shown that HBcAg can be expressed successfully using PVX- and CPMV-based vectors and that the protein retains its ability to self-assemble [58].

The expression and assembly of HBcAg were investigated in *N. benthamiana* plants using the expression system based on the deleted version of cowpea mosaic virus RNA-2. HBcAg was produced in plants at a level of 1 mg/g fresh weight and self-assembled into VLPs [59].

The production of the HBcAg dimer as a single polypeptide chain by the tandem fusion of two HBcAg open reading frames was described [60]. The dimers assembled into VLPs that could be used as scaffolds for displaying natively folded proteins on the surface of HBc particles either through genetic fusion or through noncovalent attachment [61].

### 4.2. Human Papillomavirus

One of the popular objects in the field of VLP engineering is the human papillomavirus (HPV). The great interest in papillomaviruses was due not only to their favorable structural properties but also to the need for a vaccine to prevent HPV infections. Numerous clinical trials and post-marketing surveillance have shown that HPV vaccines are safe and effective in preventing HPV infections [1]. The expression of the L1 gene alone, or L1 together with L2, is sufficient for the self-assembly of VLPs [1]. HPV proteins were expressed in different systems, including transgenic plants [62,63,64,65,66,67,68], and using transient expression. HPV L1 protein was transiently expressed in *N. benthamiana* with an efficiency of up to 20–37 mg/g fresh leaf using a tobamovirus-based vector [69]. Human-codon-optimized and chloroplast-targeted L1 protein was detected at a level of 0.5 mg/g in *N. benthamiana* plants upon transient expression. The protein assembled into higher-order structures and was highly immunogenic in mice after subcutaneous injection and elicited neutralizing antibodies [70].

The L1 gene (HPV8 type) was expressed in *N. benthamiana* using pEAQ-HT and a tobacco mosaic virus (TMV)-based replicating vector. Increased L1 gene expression was obtained when 22 amino acid residues at the C-terminus were deleted, eliminating the nuclear localization signal. Plant-produced HPV8 L1 proteins, including a truncated variant, assembled into VLPs [71]. Later, the L1 gene of an HPV16-type virus was transiently expressed in *N. benthamiana* using the pEAQ-HT vector, and the production and recovery of highly purified VLPs were reported [72].

L1 proteins of eight high-risk (HPV 16, 18, 31, 33, 35, 45, 52, and 58) and two low-risk (HPV 6 and 34) HPV types were successfully expressed in *N. benthamiana*, and transmission electron microscopy analysis showed the presence of VLPs and/or capsomeres. Immunogenicity studies were conducted in mice utilizing HPV 35, 52, and 58 VLPs and showed that type-specific anti-L1 antibodies were produced that were able to successfully neutralize homologous HPV pseudovirions in pseudovirion-based neutralization assays [73]. Pseudovirions of HPV were produced in *N. benthamiana* plants by co-infiltration of vectors expressing L1 and L2, and putative pseudovirions were purified [74].

### 4.3. Influenza Virus

The influenza virus poses a significant threat to the world’s population. The WHO estimates that annual epidemics of influenza result in ~1 billion infections, 3–5 million cases of severe illness, and 290,000–650,000 deaths [75].

Hemagglutinin (HA), the most abundant glycoprotein on the surface of the influenza virus, has become a target for recombinant vaccine development. Hemagglutinin can assemble into VLPs in plant cells even in the absence of viral envelope protein components [76]. HAs from strains A/Indonesia/5/05 (H5N1) and A/New Caledonia/20/99 (H1N1) were expressed in plants [76]. The immunization of mice with the H5 VLPs conferred complete protection from a lethal influenza A virus challenge. The assembly of HA into VLPs upon expression in *N. benthamiana* was demonstrated for several other HAs from influenza A virus, including H2, H3, H6, and H9 subtypes, and from influenza B virus [77], as well as for HA from pandemic A/H1N1 virus [77]. The rapid production of VLPs based on subtype H7 hemagglutinin in plants has also been reported [78]. More recently, the immunological properties [79,80] and structural composition [81] of HA-based VLPs produced in tobacco plants have been studied in detail.

The industrial and clinical development of an influenza VLP-based vaccine was performed by Medicago Company. HA VLPs were produced for pandemic strains such as H5N1 [50,82] and H7N9 [78]. Then, a quadrivalent VLP formulation for seasonal flu (A/California/07/2009 H1N1, A/Hong Kong/4801/2014 H3N2, B/Brisbane/60/08 and B/Phuket/3073/2013) was generated, which successfully completed three phases of clinical trials [49,83,84].

VLPs formed by the hemagglutinin of avian influenza virus A/chicken/South Africa/N2826/2016 (H6N2) were also transiently expressed in plants. A single dose of the plant-produced VLP vaccine elicited a high level of H6-specific antibodies in chickens. It has been estimated that one kilogram of plant leaf material can provide vaccines for between 5000 and 30,000 chickens, depending on the effective dose and whether one or two immunizations are administered [85].

### 4.4. Severe Acute Respiratory Syndrome-Related Coronavirus-2 (SARS-CoV-2)

Several research groups have been involved in the expression of candidate vaccines based on the S glycoprotein of SARS-CoV-2 [86,87,88].

Medicago and GlaxoSmithKline have developed a vaccine against COVID-19 (COVIFENZ). The full-length S-glycoprotein gene was expressed in *N. benthamiana* plants. The S protein was modified with R667G, R668S, and R670S substitutions at the S1/S2 cleavage site to increase stability and K971P and V972P substitutions to stabilize the protein in the pre-fusion conformation. The signal peptide was replaced with a plant signal peptide, and the transmembrane domain (TM) and cytoplasmic tail (CT) of the S protein were also replaced with TM/CT from Influenza H5 A/Indonesia/5/2005 to increase VLP assembly and budding. Self-assembled VLPs bearing S-protein trimers were isolated from the plant biomass and subsequently purified. The trimeric spike glycoproteins were displayed at the surface of the VLPs [89]. In February 2022, Health Canada authorized the use of this vaccine for preventing COVID-19 infection in adults. In February 2023, the closure of Medicago and the recall of Medicago products from the market were announced.

It was demonstrated that VLPs could be successfully obtained in plants by co-expressing three SARS-CoV-2 proteins: membrane (M), envelope (E), and nucleocapsid (N). The shape and size of the plant-produced VLPs were similar to those of native SARS-CoV-2 virus particles without the spike [86].

### 4.5. Foot-and-Mouth Disease Virus

Foot-and-mouth disease virus (FMDV) is the etiological agent of foot-and-mouth disease. A number of studies have been devoted to the development of candidate vaccines against FMDV [56]. The production of recombinant FMDV VLPs requires the simultaneous expression of the capsid protein precursor P1-2A and protease 3C, the latter of which cleaves the precursor to make the structural proteins VP0, VP3, and VP1. These proteins subsequently self-assemble to produce the viral capsid [90]. The P1-2A polyprotein and protease 3C were expressed in *N. benthamiana*. The recombinant protein yield was 3–4 µg/g of fresh leaf tissue. Both mice immunized with purified VLPs and mice immunized with the crude leaf extract elicited a specific humoral immune response with similar antibody titers [91].

### 4.6. Poliovirus

Poliovirus (PV) is the causative agent of polio, also known as poliomyelitis. PV P1 protein is processed by the viral proteinase 3CD to produce three capsid proteins. P1 regions of either the wild-type PV3 serotype or the mutant PV3, identified as having stabilizing mutations within the coat proteins [92], were produced along with 3CD proteinase in plants. The yields in the purified samples were about 0.06 mg/g fresh weight for the mutant PV3 particles (sVLP) and 0.04 mg/g fresh weight for wild-type PV3 VLPs. Structural analysis of sVLP at 3.6 Å resolution by cryo-electron microscopy and single-particle reconstruction revealed a structure almost indistinguishable from wild-type PV3. The immunization of mice carrying the gene for the human PV receptor with plant-produced sVLPs elicited similar neutralizing antibody responses to the inactivated vaccine and protected animals from a challenge with a virulent virus at levels similar to those observed in the case of the inactivated vaccine [93].

### 4.7. Dengue Virus

Dengue virus (DENV) is the cause of dengue fever, which is transmitted by mosquitoes. The production of DENV VLPs was performed in *N. benthamiana* using transient expression. Co-expression of DENV structural proteins and a truncated version of the non-structural proteins lacking NS5, which encodes RNA-dependent RNA polymerase, resulted in the assembly of DENV VLPs in plants. The yield of VLPs after purification was about 2 µg/g fresh weight. Immunogenicity assays in BALB/c mice revealed that plant-made VLPs induced a specific antibody response in mice [94].

### 4.8. Hepatitis E Virus

Hepatitis E virus (HEV) is a causative agent of acute hepatitis, mainly transmitted by the fecal–oral or zoonotic route [29]. The truncated capsid protein of HEV genotype 3, encoded by ORF2 and consisting of a.a. residues 110–610, was expressed in *N. benthamiana* plants using the pEAQ-HT system and the self-replicating vector pEff [29]. The vector pEff provided expression levels up to 10% of the soluble protein fraction (~0.3 mg/g fresh weight). The recombinant protein formed nanosized VLPs. The immunization of mice with the plant-produced protein induced high levels of HEV-specific antibodies in serum [95].

### 4.9. Rotavirus

Rotaviruses (RVs) are the most common cause of diarrheal illness among infants and young children [96]. The highly conserved and abundant structural protein VP6 of RVs is an immunogen that is capable of self-assembling into nanosized structures [97]. The VP6 protein was expressed in *N. benthamiana* plants using PVX-based vectors either as a fusion with the PVX coat protein or from an additional subgenomic RNA promoter inserted to allow the independent expression of both VP6 and the PVX coat protein. Both approaches yielded VP6, which retained the ability to form trimers [98].

VLPs composed of the viral proteins VP7, VP6, and VP2 of the G1 genotype were produced in *N. benthamiana*. The obtained VLPs were structurally similar to triple-layered rotavirus particles. Two doses of aluminum hydroxide-adjuvanted VLPs, administered intramuscularly, elicited a robust homotypic neutralizing antibody response in rats. In rabbits injected with these VLPs four times intramuscularly with aluminum hydroxide adjuvant, no significant toxicity was observed [99].

### 4.10. Norovirus

Norovirus (NoV) infection is the leading cause of acute gastroenteritis worldwide [100], with GII.4 norovirus strains responsible for most outbreaks [101]. The capsid protein of norovirus was transiently expressed in *N. benthamiana* by exploiting different expression vectors.

The expression of norovirus GII.4 capsid in plants using the magnICON system resulted in the rapid onset of cell death and, correspondingly, a rather low protein yield of 0.3 mg/g fresh weight. Transmission electron microscopy of plant-derived proteins confirmed the presence of fully assembled VLPs. Partially purified VLPs were used to immunize mice by intranasal delivery and generated specific mucosal and serum antibody responses [102].

The use of a replicating vector based on the geminivirus BeYDV resulted in expression levels of the NoV capsid protein (GII.4) up to 2.3 mg/g [103].

The MagnICON platform was also applied for the production of Norwalk virus VLPs. VLPs were produced at a level of 0.86 mg/g fresh weight (12 dpi) and provided immune responses in mice [104].

### 4.11. Rift Valley Fever Virus

Rift Valley fever virus (RVFV) is an emerging mosquito-borne virus and hemorrhagic fever agent. The transmembrane domain of the RVFV Gn protein was replaced with that of HA from the H5N1 avian influenza virus strain, and the hybrid protein was transiently expressed in *N. benthamiana* with a final yield of about 57 µg/g fresh weight. The protein formed VLPs of 49–60 nm in size, which were shown to elicit a specific antibody response to the Gn protein in mice [105].

### 4.12. Beak and Feather Disease Virus

Psittacine beak and feather disease, caused by beak and feather disease virus (BFDV), is a threat to endangered psittacine species. The full-length BFDV coat protein (Cap) and a truncated Cap (ΔN40) were transiently expressed in *N. benthamiana* as fusions to elastin-like polypeptide [106]. Plant-produced full-length BFDV Cap was assembled into VLPs, yielding less than 5 µg/g of fresh weight [107].

### 4.13. Porcine Circovirus 2

Porcine circovirus (PCV2) causes enormous economic losses to the swine industry worldwide. The capsid protein (Cap) of PCV2 was expressed in *N. benthamiana* using the pEAQ-HT vector and purified using sucrose gradient ultracentrifugation. The Cap self-assembled into VLPs resembling native virions, and up to 6.5 mg of VLPs could be purified from 1 kg of leaf biomass. The mice immunized with the plant-produced PCV2 VLPs elicited specific antibody responses to the PCV2 Cap [108].

Another study demonstrated the transient expression of the PCV2 Cap in *N. benthamiana* and the purification of the hexahistidine-tagged PCV2 VLPs by affinity chromatography, with a yield of 102 µg/g plant leaves. Electron microscopy confirmed that purified Cap self-assembled into VLPs, which were shown to induce a strong immune response in guinea pigs [109].

### 4.14. Atlantic Cod Nervous Necrosis Virus

VLPs of a fish virus, ACNNV, were successfully produced by the transient expression of the coat protein in *N. benthamiana* plants. Using the pEAQ-HT system, a yield of up to 10 µg/g fresh weight was obtained. The administration of the plant-produced VLPs to sea bass (*Dicentrarchus labrax*) showed that they could protect the fish against a subsequent virus challenge [110].

### 4.15. Bluetongue Virus

Bluetongue virus (BTV) causes a severe disease in ruminants, notably sheep and cattle, causing, among other symptoms, facial swelling, lameness, and infertility, leading to mortality in some cases. BTV VLPs, formed by four distinct proteins (VP2, VP3, VP5, and VP7), were produced in *N. benthamiana* using the pEAQ-HT expression vector with a yield of 70 µg/g. The obtained VLPs were shown to elicit a strong antibody response in sheep and provided protective immunity against a challenge with a South African BTV-8 field isolate [111]. The co-expression of BTV-8 serotype VP2, VP3, VP5, and VP7 proteins was performed in *N. benthamiana* using pEAQ-HT and BeYDV-based vectors [112].

Combinations of BTV capsid proteins from more than one serotype were expressed in *N. benthamiana* plants and assembled to form chimeric BTV-3 and BTV-4 VLPs. The assembled homogeneous BTV-8 serotype, as well as chimeric BTV-3 (BTV-8 proteins VP3 and VP7, and BTV-3 proteins VP5 and VP2) and chimeric BTV-4 (BTV-8 proteins VP3 and VP7, and BTV-4 proteins VP2 and VP5; BTV-8 protein VP3 and BTV-4 proteins VP2, VP5, and VP7) VLP serotypes, was confirmed. The yield of BTV-3 VLPs was 26 mg/g of plant leaf tissue. A conservative estimate is that 570 sheep could be vaccinated with partially purified chimeric VLPs obtained from one kilogram of plant leaf tissue. Plant-produced chimeric BTV-3 and BTV-4 VLPs were both able to induce long-lasting serotype-specific neutralizing antibodies equivalent to those obtained for control monovalent live-attenuated vaccine [113].

### 4.16. African Horse Sickness Virus

African horse sickness is a highly infectious disease of domestic equids in Africa caused by African horse sickness virus (AHSV). The formation of virus-like particles requires the simultaneous expression of four different proteins (VP2, VP3, VP5, and VP7), as in the case of the Bluetongue virus. The expression and assembly of AHSV serotype 5 VLPs were achieved in *N. benthamiana*. Antibodies raised in guinea pigs upon immunization were shown to neutralize the live virus in cell-based assays [114].

The safety and immunogenicity of plant-derived AHSV virus-like particles have also been demonstrated in horses [115]. Another study demonstrated that the transient co-expression of the four AHSV capsid proteins allowed the assembly of homogeneous AHSV-1 VLPs, as well as single, double, and triple chimeric VLPs in which one capsid protein was derived from one AHSV serotype and at least one other capsid protein was derived from another AHSV serotype. The safety and immunogenicity of the plant-produced triple chimeric AHSV-6 VLPs were confirmed in horses [116]. It was also demonstrated that insoluble AHSV-5 VP7 quasicrystals produced in *N. benthamiana* were immunogenic and induced both humoral and cell-mediated responses in guinea pigs [117].

### 4.17. Infectious Bursal Disease Virus

Infectious bursal disease is an acute, highly contagious, immunosuppressive disease of chickens caused by infectious bursal disease virus (IBDV), which critically threatens the development of the global chicken industry and causes huge economic losses [118]. IBDV major capsid protein VP2 was transiently expressed in plants. A mixed population of differently shaped particles, ranging from spherical capsids, with diameters between ~25 and ~70 nm, to tubular structures, with variable lengths from 100 to 400 nm, was revealed. Intramuscular immunization of chickens with these putative VLPs induced the production of specific anti-IBDV antibodies in titers comparable to those induced by the commercial vaccine. Moreover, all the immunized birds survived a challenge with a highly virulent IBDV strain [119].

### 4.18. Cottontail Rabbit Papillomavirus

Cottontail rabbit papillomavirus (CRPV) provides a robust model to study viral interaction with the host and progression to cancer, as well as for viral vaccine research. The CRPV L1 capsid protein gene was expressed in transgenic plants and transiently via a TMV-based vector in *N. benthamiana*. The amount of CRPV L1 produced ranged from 0.4 to 1 µg/g in transgenic plants and from 0.15 to 0.6 µg/g of total leaf biomass via transient expression. The protein did not assemble into detectable VLPs; however, immunoelectron microscopy showed presumptive pentamer aggregates, and the extracted protein reacted with conformation-specific and neutralizing monoclonal antibodies. Vaccinated rabbits were protected from developing warts when subsequently challenged with the live virus [120].

### 4.19. Bovine Papillomavirus 1

Bovine papillomavirus 1 (BPV1) is an important causative agent of economically damaging bovine papillomas in cattle and equine sarcoids in horses and wild equids. BPV1 capsid proteins L1 and L1/L2 were transiently expressed in *N. benthamiana* to produce VLPs and pseudovirions [121]. BPV1 L1 was expressed with a yield of 183 µg/g fresh leaf tissue and self-assembled into VLPs that elicited a highly specific and potent immune response in rabbits [122].

### 4.20. Infectious Bronchitis Virus

Infectious bronchitis is a highly contagious, acute respiratory disease in chickens, with a severe economic impact on poultry production globally [123]. Plants were used to produce a VLP vaccine based on a modified full-length spike (S) protein of the QX-like IB variant of infectious bronchitis virus (IBV). It has been calculated that 1 kg of plant leaf material is sufficient to produce at least 3352 individual 5 µg doses of the VLP vaccine [124]. In a study using a homologous live IB QX-like virus, VLP-vaccinated birds induced specific antibodies to the S protein at levels comparable to those induced in live-vaccinated birds. The VLP-vaccinated birds had reduced oropharyngeal and cloacal viral shedding compared to an unvaccinated challenged control and were more protected against tracheal ciliostasis than the live-vaccinated birds [124,125].

### 4.21. Piscine Myocarditis Virus

Cardiomyopathy syndrome (CMS) is a severe cardiac disease occurring in the grow-out sea phase of farmed Atlantic salmon. The putative capsid protein of piscine myocarditis virus (PMCV) was expressed in *N. benthamiana* and formed VLPs. A total of 1.2 mg of antigen could be isolated from 250 g of infiltrated leaf material [126].

A summary of studies aimed at developing vaccines based on VLPs formed by capsid proteins of the target virus is presented in Table 1.

## 5. Plant-Produced Vaccines Based on Chimeric VLPs

### 5.1. Hepatitis B Core Antigen as a Carrier for Foreign Antigens

The use of HBc antigen as a carrier VLP displaying foreign epitopes and other peptides was first reported in 1987 [127]. Since then, HBc VLPs have been widely utilized in nanobiotechnology to display antigens and/or cell-targeting signals and to package poly- and oligonucleotides [1].

**Figure 5:**
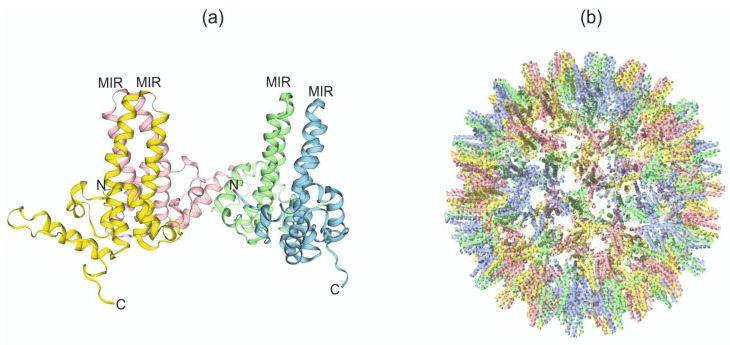
The structures of HBc (PDB 6HU4). (**a**) Monomer chains A, B, C, and D are marked in blue, green, pink, and yellow, respectively. Three-dimensional modeling was performed by SWISS-MODEL [130]. (**b**) VLPs of HBc [131].

The full-length HBc protein was used as the initial platform for foreign antigen presentation, and truncated variants, mainly HBc 1–144 or HBc 1–149, were also used, as they were found to be necessary and sufficient to serve as icosahedral scaffolds for proper self-assembly of VLPs [128]. Three regions of HBc, namely, the N-terminus, the major immunodominant region (MIR) around the protruding region 78–82 on the tip of the spike, and the C-terminus, can accommodate foreign insertions while still allowing the correct self-assembly of modified HBc monomers into chimeric VLPs (Figure 5) [129].

Covalent conjugation using the SpyTag/SpyCatcher system in vivo in *N. benthamiana* cells was demonstrated by an example of VLPs formed by two tandem copies of HBc (tHBc) and the model antigen GFP. It was shown that tHBc VLPs could be successfully conjugated with GFP in the cytosol and ER without altering VLP formation or GFP fluorescence. The final yield of purified unconjugated tHBc and cytosolic tHBc-SC + GFP-ST was approximately 150 μg/g leaf tissue, whereas ER-targeted tHBc-SC + GFP-ST had a final yield of approximately 6 μg/g. Successful conjugation of tHBc VLPs to the HIV capsid protein P24 in the cytosol was also demonstrated [61].

VLPs formed by tHBc were employed to expose the capsular polysaccharide of *Burkholderia thailandensis* using chemical conjugation to develop a vaccine against melioidosis. tHBc VLPs were produced in *N. benthamiana* leaves, and the capsular polysaccharide was isolated from non-pathogenic *B. thailandensis* and conjugated to carrier proteins by reductive amination. The conjugated vaccine was able to protect mice against an intraperitoneal challenge with *B. pseudomallei* [132].

The consensus sequence of the antigenic domain of dengue virus glycoprotein III (cEDIII) was inserted into HBc MIR and produced in *N. benthamiana*. The yield of purified VLPs was in the range of ∼12–16 µg/g. Mice immunized with the chimeric VLPs showed positive seroconversion to the cEDIII antigen [133].

The HPV minor capsid protein L2, displayed on the surface of HBc particles, was produced in plants. This vaccine candidate was found to be highly immunogenic in mice [134].

Zika virus (ZIKV) is considered a global public health threat due to its rapid spread and its association with neonatal complications [135]. To generate HBc VLPs bearing the Zika virus envelope domain III (ZE3), the ZE3 antigen (amino acids 301–406) flanked by flexible linkers was inserted into the second of two tandem copies of HBc. Alternatively, ZE3 was fused at the C-terminus to an HBc monomer. Both fusion proteins were expressed in *N. benthamiana* and assembled into highly immunogenic VLPs [136].

In another study, the ZE3 antigen (303–403 a.a.) was genetically fused to the C-terminus of HBc, and chimeric VLPs were produced in *N. benthamiana* plants with an average accumulation of 1.824 mg/g leaf fresh weight, representing a very high expression level of recombinant proteins in plants. The chimeric HBc particles were shown to be highly immunogenic, as two doses elicited strong humoral and cellular responses in mice [137].

West Nile virus (WNV) is neurotropic and can infect the central nervous system of humans and animals [138]. HBc particles displaying the WNV envelope protein domain III at the C-terminus were rapidly produced in *N. benthamiana* plants and achieved high expression levels of approximately 1.2 mg/g fresh leaf weight. The chimeric VLPs were highly immunogenic and elicited potent humoral responses in mice [139].

HBc was used to display the peptide epitope (551–607 aa) from the hepatitis E virus ORF2 capsid protein, which was inserted into the HBc MIR. The fusion protein was expressed in *N. benthamiana*, formed VLPs, and was recognized by anti-HBcAg antibodies and anti-HEV IgG-positive porcine serum. The yield of VLPs was approximately 10 µg/g leaf biomass [140].

A highly conserved influenza M2e peptide was fused to the N-terminus of HBc. The fusion protein was expressed in *N. benthamiana* in amounts reaching 5–10% of the total soluble protein and formed VLPs with the M2e peptide displayed on the surface [23]. Experiments in mice showed the high immunogenicity of plant-produced M2eHBc particles and their protective effect against a lethal influenza challenge [141].

A summary of studies aimed at the development of HBc-based vaccine candidates produced in plants is presented in Table 2.

### 5.2. Hepatitis E Virus Coat Protein as a Carrier of Foreign Antigens

VLPs based on a truncated coat protein of HEV and carrying the M2e peptide (one or four copies) of influenza A virus or the receptor-binding domain (RBD) of the SARS-CoV-2 spike glycoprotein were obtained in *N. benthamiana*. The fusion proteins HEV CP/M2e and HEV CP/4M2e were expressed at levels of about 300–400 μg/g and 150–200 μg/g per fresh leaf tissue with purification yields of 200 μg/g and 60–80 μg/g, respectively. The fusion protein HEV CP/RBD was expressed at about 80–100 μg/g; the yield after purification was up to 20 μg/g. The recombinant proteins formed nanosized VLPs that could be recognized by antibodies against the inserted epitopes. ELISA showed that antibodies of COVID-19 patients can bind plant-produced HEV CP/RBD virus-like particles [29,30,142].

### 5.3. The L1 Capsid Protein of Human Papillomavirus as a Carrier of the M2e Peptide of Influenza A Virus

The L1 protein of HPV type 16 was used as a carrier of two antigens of influenza A virus: the M2e peptide or its shorter version (2–9 aa) containing a highly conserved N-terminal epitope that is common to influenza M1 and M2 proteins. The fusion proteins were expressed in *N. benthamiana* using the pEAQ-HT system, with yields ranging from 30 to 120 μg/g. The fusion proteins were recognized by a panel of linear and conformation-specific anti-HPV-16 L1 monoclonal antibodies, and two of them also reacted with anti-influenza monoclonal antibodies. Electron microscopy showed that the fusion proteins produced in plants assembled into higher-order structures such as VLPs with T = 1 or T = 7 symmetry or capsomeres [143].

### 5.4. Bluetongue Virus VP3 Protein as a Carrier for Envelope Protein Domain III of Dengue Viruses DENV1 and DENV4 and Zika Virus

The EIII ectodomain of DENV1, DENV4, and Zika viruses was displayed on the inner surface of bluetongue virus core-like particles (B-CLPs) by fusing the antigen at the N-terminus of bluetongue VP3 protein. Recombinant proteins were produced in *N. benthamiana*. B-CLPs yielded 5–15 μg/g fresh weight after only one ultracentrifugation step and one buffer exchange/concentration step. EDIII integration did not prevent the self-assembly of chimeric B-CLPs [94].

### 5.5. Alfalfa Mosaic Virus Coat Protein as a Carrier of Plasmodium Falciparum Pfs25 Protein

The AMV-derived malaria vaccine candidate was based on the Pfs25 protein, a key transmission-blocking vaccine antigen. The 23–193 amino acid region of P. falciparum Pfs25 protein was linked to the N-terminus of the AMV coat protein and produced in *N. benthamiana* plants using a TMV-based vector. Infiltrated plants accumulated the recombinant protein with peak levels of approximately 50 μg/g fresh weight. The immunization of mice with one or two doses of Pfs25-AMV particles plus Alhydrogel adjuvant induced serum antibodies exhibiting complete transmission-blocking activity over the six-month study period [144].

In a phase 1 study, this vaccine candidate (Pfs25 VLP-FhCMB) was shown to be generally safe in healthy volunteers. However, although the vaccine induced Pfs25-specific antibodies in a clinical trial, limited inhibition of parasite transmission to mosquitoes was observed, indicating the need for improved vaccine formulations [145].

### 5.6. Bacteriophage AP205 Capsid Protein as a Carrier for West Nile Virus Envelope Protein Domain III (WNV EDIII)

A candidate vaccine against WNV was obtained using the SpyTag/SpyCatcher (ST/SC) conjugation system. WNV envelope protein domain III (EDIII), containing specific epitopes, was fused to and displayed on phage AP205 virus-like particles after the separate production of WNV-EDIII and AP205 coat proteins in *N. benthamiana*. The yield of the purified WNV-EDIII protein was calculated to be in the range of 33.5–69 μg/g fresh weight, while the yield of purified AP205:EDIII VLPs was about ~36 μg/g. Subcutaneous immunization of mice with 5 μg of purified AP205:EDIII VLPs elicited a potent IgG response to WNV EDIII [146].

### 5.7. Spherical Nanoparticles Derived from Tobacco Mosaic Virus as an Epitope Presentation Platform

TMV has proven to be one of the most promising expression vectors and a popular antigen carrier, and it has been used in a number of studies [1]. A recent advance in this field has been the development of a new TMV-based nanoplatform, the so-called spherical nanoparticles (SNPs). SNPs were obtained by the two-step thermal remodeling of native TMV virions, where irregularly shaped and sized particles were obtained by heating at 90 °C and then converted to SNPs by heating at 94 °C [147,148]. Foreign proteins could be bound to the surface of SNPs in vitro, which was first shown for GFP. Later, Karpova et al. [149] obtained SNPs containing one of the following foreign antigens: antigenic determinant A of rubella virus glycoprotein E1, recombinant protein containing the M2e peptide of influenza A virus, a recombinant antigen consisting of three epitopes of influenza A virus hemagglutinin, PVX coat protein, PVX coat protein fused with an epitope of plum pox virus (PPV) coat protein, and bovine serum albumin (BSA). “Mixed” compositions were also obtained by binding two different foreign antigens to SNPs. The assembly procedure involved short-term incubation of the SNP with the foreign protein of interest and relied on noncovalent interactions such as electrostatic and hydrophobic bonds [148,149,150,151]. This technology has also been applied to develop vaccines against rotavirus [152], SARS-CoV-2 [153,154], and anthrax [155,156].

A summary of studies aimed at the development of chimeric VLP vaccines in plants is presented in Table 3.

## 6. Conclusions and Perspectives

The use of virus-like particles in vaccine development is a rapidly growing area. The ability of virus-like particles to self-assemble has made them an attractive platform for vaccine development. VLPs derived from human and animal viruses serve as independent vaccines to protect against the viruses from which they were derived. VLPs from animal, plant, and bacterial viruses can be used to display foreign peptides to enhance the immunogenicity of peptides derived from other infectious agents to protect against these agents. VLPs are able to stimulate both the innate and adaptive immune systems, and in some cases, VLP-based vaccines can be used without any adjuvants.

Several recombinant expression systems with different efficiencies have been applied for VLP production [2,3,4,5,157,158,159]. Plants are a promising platform for VLP production. The rapid mass production of VLPs in plants can be achieved by using a viral vector-based transient expression system. The progress of Medicago’s CoVLP vaccine has established the plant as an effective production platform for rapid, robust, safe, and economical vaccines. Biopharmaceutical companies are expressing interest in the large-scale production of VLPs for vaccine development.

At the same time, the use of transient expression systems for the production of recombinant proteins, and in particular VLPs, in plants has revealed several problems. One major problem is the unpredictability of the expression level that can be achieved for a particular target protein. Despite a large number of studies, it remains unclear to date which properties of a protein (and/or the gene encoding it) limit the efficiency of its production using a given expression vector. Therefore, in many cases, it is necessary to test various vectors and different designs of recombinant proteins. Another problem is the difference in the protein glycosylation system in plants and mammals, which can affect the biological activity of proteins obtained in plants. A way to solve this problem may be to use plants with modified glycosylation pathways.

Overall, plant-produced VLP-based vaccines are of great interest and are promising areas of vaccine development. Regarding the prospects of plant-produced VLPs, besides the interest of biopharmaceutical companies in the large-scale production of VLPs for vaccine development, further investigations are necessary on various aspects, especially on easy and low-cost VLP purification, as well as on scaling the technology from the laboratory level to industrial production. At the same time, plant-derived VLPs must be competitive, since the main stumbling block in this process is the financial sustainability of biopharmaceutical companies.

## Figures and Tables

**Table 1:** “Natural” vaccine candidates based on plant-produced VLPs.

Vaccine Target	Composition of the VLP	Expression Vector	Efficiency, per Fresh Weight	Reference
Hepatitis B virus	HBcAg	MagnICON	2380 μg/g	[57]
	HBcAg	PVX-based	N/a	[58]
	HBcAg	CPMV-based	N/a	[59]
	HBcAg	pEAQ-HT	1000 μg/g	[59]
	tandem HBcAg	pEAQ-HT	200 μg/g	[60]
Human papillomavirus	HPV16 L1	TMV-based	20–37 μg/kg *	[69]
	HPV16 L1	BeYDV-based	533 μg/g	[70]
	HPV8 L1	pEAQ-HT	60 μg/g	[71]
	HPV8 L1∆C22	pEAQ-HT	240 μg/g	[71]
	HPV8 L1	MagnICON	3.5 μg/g	[71]
	HPV8 L1∆C22	MagnICON	17 μg/g	[71]
	HPV16 L1	pEAQ-HT	N/a	[72]
	L1 (HPV 16, 18, 31, 33, 35, 45, 52, 58); HPV 6 and 34	BeYDV-based	N/a	[73]
	HPV16 L1, L2	BeYDV-based	N/a	[74]
Influenza virus	HA (H1N1)	N/a	N/a	[76]
	HA (H5N1)	N/a	N/a	[76]
	HA of H2, H3, H6, H9, H1N1	N/a	N/a	[77]
	HA of H7	N/a	N/a	[78]
	HA H5N1 (Medicago)	N/a	N/a	[50]
	HA H7N9 (Medicago)	N/a	N/a	[78]
	quadrivalent HA VLP	2X35S/CPMV-HT	N/a	[78,84]
	HA of H6	pEAQ-HT	95 μg/g *	[85]
SARS-CoV-2	S glycoprotein		N/a	[89]
	co-expression of M, E, and N	non-viral	N/a	[86]
Foot-and-mouth disease Virus	P1-2A and 3C	BeYDV-based pEAQ-HT	3–4 μg/g *	[91]
Poliovirus	P1 and 3CD	pEAQ-HT	0.06 μg/g *	[93]
Dengue virus	DENV SP and NSPs, lacking NS5	pEAQ-HT	2 μg/g *	[94]
Hepatitis E virus	HEV ORF 2 (110–610 aa)	pEAQ-HT pEff	N/a 300 μg/g	[95]
Rotavirus	VP6	PVX-based	50 μg/g	[98]
	VLPs composed of VP7, VP6, and VP2 of G1	CPMV HT	4.9 μg/g *	[99]
Norovirus	CAP	MagnICON	280 μg/g	[102]
Norovirus	CAP (GII.4)	BeYDV-based	1000 μg/g	[103]
	CAP (GI)	BeYDV-based	2300 μg/g	[103]
Norwalk virus	CP	MagnICON	800 μg/g	[104]
Rift Valley fever virus	Gn replaced with HA from H5N1	pEAQ-HT	57 μg/g *	[105]
Beak and feather disease virus	Cap and Cap (ΔN40)	BeYDV-based	<5 μg/g *	[106]
Porcine circovirus 2	Cap	pEAQ-HT	6.5 μg/g *	[108]
	Cap	non-viral	102 μg/g *	[109]
Atlantic cod nervous necrosis virus	CP	pEAQ-HT	10 μg/g *	[110]
Bluetongue virus	BTV-8 VP2, VP3, VP5, and VP7	pEAQ-HT	70 μg/g *	[111]
	BTV-8 VP2, VP3, VP5, and VP7	pEAQ-HT and BeYDV-based	N/a	[112]
	Chimeric BTV-3 and BTV-4 VLPs	pEAQ-HT	26 μg/g *	[113]
African horse sickness virus	AHSV serotype 5 VP2, VP3, VP5, and VP7	pEAQ-HT	N/a	[114]
	AHSV chimeric VP2, VP3, VP5, and VP7	pEAQ-HT	16 μg/g *	[116]
	AHSV-5 VP7 quasicrystals	BeYDV-based	N/a	[117]
Infectious bursal disease virus	CP VP2	non-viral	N/a	[119]
Cottontail rabbit papillomavirus	L1 protein	TMV-based	0.15–0.6 μg/g	[120]
Bovine papillomavirus 1	BPV L1 and L1/L2	BeYDV-based	N/a	[121]
	BPV1 L1	pEAQ-HT	183 μg/g *	[122]
Infectious bronchitis virus	S	pEAQ-HT	17 μg/g *	[124,125]
Piscine myocarditis virus	putative CP	pEAQ-HT	4.8 μg/g *	[126]

* Purification yield. N/a—information not available.

**Table 2:** HBcAg-based vaccine candidates produced in plants.

Vaccine Target	HBcAg Composition	Epitope	Insertion Position	Expression Vector	Efficiency, per Fresh Weight	Reference
Dengue virus	Tandem Hbc	EDIII, 103 a.a.	MIR	pEAQ-HT	12–16 μg/g *	[133]
Human papillomavirus	Tandem Hbc	L2, 14–122 a.a.	MIR	BeYDV-based	>3 mg **	[134]
Zika virus	Tandem Hbc	EDIII, 301–406 a.a.	MIR	BeYDV-based		[136]
Zika virus	HBcΔ	EDIII, 301–406 a.a.	C	BeYDV-based		[136]
Zika virus	HBcΔ	EDIII, 303–403 a.a.	C	MagnICON	1824 μg/g	[137]
West Nile virus	HBcΔ	EDIII, 296–415 a.a.	C	MagnICON	1200 μg/g	[139]
Hepatitis E virus	HBc	ORF2 551–607 a.a.	MIR	pEAQ-HT	10 μg/g *	[140]
Influenza A virus	HBc	M2e, 2–24 a.a.	N	pEff	10% of the total soluble protein	[141]

* Purification yield; ** from a single plant leaf.

**Table 3:** Chimeric VLP-based vaccine candidates produced in plants.

VLP Carrier	Vaccine Target	Epitope	Expression Vector	Efficiency, per Fresh Weight	Reference
Hepatitis E virus coat protein	Influenza A virus	M2e, human	pEAQ-HT, pEff	300–400 µg/g	[29]
	Influenza A virus	4xM2e, swine	pEff	150–200 µg/g	[30]
	SARS-CoV-2	RBD, 319–524 a.a.	pEff	80–100 µg/g	[142]
Human papillomavirus L1 protein	Influenza A virus	M2e, 2–24 a.a.; M2e, 2–9 a.a.	pEAQ-HT	30–120 µg/g	[143]
Bluetongue virus VP3 protein	Dengue viruses and Zika virus	EDIII	pEAQ-HT	5–15 µg/g *	[94]
Alfalfa mosaic virus coat protein	Malaria	Pfs25	TMV-based	50 µg/g	[144]
Bacteriophage AP205 capsid protein	West Nile virus	EDIII	pEAQ-HT BeYDV-based	36 µg/g *	[146]

* Purification yield.

## Data Availability

The data are available in the article.

## Supplementary Materials

The following supporting information can be downloaded at www.mdpi.com/article/10.3390/plants13243564/s1: Table S1: Approved VLP-based vaccines against infectious diseases [160,161,162,163,164,165,166,167,168,169,170,171,172,173,174,175,176,177,178,179,180].
