# Supplementary material for: Virus-like Particles Produced in Plants: A Promising Platform for Recombinant Vaccine Development
Source: Plants (Basel). 2024 Dec 20;13(24):3564. doi: 10.3390/plants13243564 (PMC11678810; doi:10.3390/plants13243564)
Supplement: Supplementary file 1 [file plants-13-03564-s001.zip › Table S1. Approved VLP-based vaccines against infectious diseases.pdf]

Table S1. Approved VLP-based vaccines against infectious diseases.

System	Organism	Infection	Antigen	Trade name	Company	Reference
Bacteria	*E.coli*	Hepatitis E virus	368-606 aa of the HEV ORF2 capsid protein (genotype 1)	Hecolin®	Xiamen Innovax Biotec (License in China)	[163,164]
Insect	*Spodoptera frugiperda (Sf9)*	Human Norwalk Virus	capsid protein (genotype GI.1)	no trade name	Takeda	[165]
	Trichoplusia ni (Tn5)	Human Papilloma Virus	L1 protein (Types 16, 18)	Cervarix®	GlaxoSmithKline	[166]
Yeast	*S. cerevisiae*	Human Papilloma Virus	L1 protein (Types 6, 11, 16, 18)	Gardasil®	Merck	[167]
	S. cerevisiae	Human Papilloma Virus	L1 protein (Types 6, 11, 16, 18, 31, 33, 45, 52, 58)	Gardasil9®	Merck	[168]
	S. cerevisiae	Hepatitis B virus	HBsAg	Engerix-B®	GlaxoSmith Kline (GSK)	[169]
	P. pastoris	Hepatitis B virus	HBsAg	Enivac HB	Panacea Biotec	[170]
	H. polymorpha	Hepatitis B virus	HBsAg	Gene VacB®	Serum Institute of India	[171]
	S. cerevisiae	Hepatitis B virus	HBsAg	Recombivax HB®	Merck	[172]
	P. pastoris	Hepatitis B virus	HBsAg	Revac-B +™	Bharat Biotech International	[173]
	P. pastoris	Hepatitis B virus	HBsAg	ShanvacTM-B	Shantha Biotechnics	[174]
	S. cerevisiae	Hepatitis B virus	HBsAg	Euvax B	LG Life Sciences	[175]
	P. pastoris	Hepatitis B virus	HBsAg	Heberbiovac HB	CIGB – Heber Biotec	[174]
	H. polymorpha	Hepatitis B	HBsAg	HepavaxGene	Crucell	[176]

		virus		®		
	P. pastoris	Hepatitis B virus	HBsAg	DTP-HEP B	P.T. Bio Farma	[177]
	S. cerevisiae	Malaria	*P. falciparum* CSP fused to the HBsAg	Mosquirix™	GSK and WRAIR	[178]
Mammalian cells	CHO	Hepatitis B virus	HBsAg , Pre-S1, and Pre-S2	Sci-Bvac™	METU and Bilkent University, Nobel Pharmaceuticals	[179]
	CHO	Hepatitis B virus	HBsAg	Bio-Hep-B	BTG (SciGen, FDS Pharma)	[180]
	CHO	Hepatitis B virus	PreS1 + 2 and HBsAg	GenHevac B	Pasteur-Merieux Aventis	[181]
Cell free		Hepatitis A virus	Inactivated HAV RG-SB	Epaxal	Crucell	[182]
		Influenza	A (H1N1), A (H3N2), B, HA, NA	Inflexal V	Crucell	[183]
